# Synaptic Connections of the Neurokinin 1 Receptor-Like Immunoreactive Neurons in the Rat Medullary Dorsal Horn

**DOI:** 10.1371/journal.pone.0023275

**Published:** 2011-08-17

**Authors:** Jian Qi, Hua Zhang, Jun Guo, Le Yang, Wen Wang, Tao Chen, Hui Li, Sheng-Xi Wu, Yun-Qing Li

**Affiliations:** 1 Department of Anatomy and K. K. Leung Brain Research Centre, The Fourth Military Medical University, Xi'an, China; 2 Department of Physiology, The Fourth Military Medical University, Xi'an, China; 3 Undergraduate Student of the 2007 in Pharmacology, The Fourth Military Medical University, Xi'an, China; University of North Dakota, United States of America

## Abstract

The synaptic connections between neurokinin 1 (NK1) receptor-like immunoreactive (LI) neurons and γ-aminobutyric acid (GABA)-, glycine (Gly)-, serotonin (5-HT)- or dopamine-β-hydroxylase (DBH, a specific marker for norepinephrinergic neuronal structures)-LI axon terminals in the rat medullary dorsal horn (MDH) were examined under electron microscope by using a pre-embedding immunohistochemical double-staining technique. NK1 receptor-LI neurons were observed principally in laminae I and III, only a few of them were found in lamina II of the MDH. GABA-, Gly-, 5-HT-, or DBH-LI axon terminals were densely encountered in laminae I and II, and sparsely in lamina III of the MDH. Some of these GABA-, Gly-, 5-HT-, or DBH-LI axon terminals were observed to make principally symmetric synapses with NK1 receptor-LI neuronal cell bodies and dendritic processes in laminae I, II and III of the MDH. The present results suggest that neurons expressing NK1 receptor within the MDH might be modulated by GABAergic and glycinergic inhibitory intrinsic neurons located in the MDH and 5-HT- or norepinephrine (NE)-containing descending fibers originated from structures in the brainstem.

## Introduction

The medullary dorsal horn (MDH) receives inputs from small-diameter thin-myelinated Aδ and unmyelinated C fibers that convey preferentially nociceptive information from the orofacial structures mainly through cranial nerves, such as trigeminal (V), facial (VII), glossopharyngeal (IX) and vagus (X) nerves [1]. Within the superficial laminae (laminae I and II) of the MDH, the primary afferent inputs terminate onto projection neurons and interneurons that send their axons outside and within the nucleus, respectively, to participate in the integration and transmission of nociceptive information from orofacial region to higher brain centers [2], [3], [4]. Glutamate is the main excitatory substance released by primary afferents. Primary afferent terminals contain excitatory substances, including pain related neuropeptides, such as substance P (SP) and calcitonin gene-related peptide (CGRP), which contribute to the orofacial nocieciptive processing [4], [5]. The initial integrative processing of nociceptive information in both spinal and medullary dorsal horns also involves inhibitory local circuits, in which γ-aminobutyric acid (GABA) and glycine (Gly) play fundamental roles as neurotransmitters [6], [7]. Local inhibitory interneurons in the dorsal horn are critical for controlling the excitability at the segmental level and thus determine how nociceptive information is relayed to higher brain structures [8]. Many studies have demonstrated that these inhibitory interneurons play essential roles in modulating the nociceptive transmission in the dorsal horn [6], [9], [10]. In addition, the superficial laminae of the dorsal horn receive dense descending inputs, including serotoninergic and norepinephrinergic systems, originating from the rostral ventromedial medulla (RVM), including the nucleus raphe magnus (NRM) and its surrounding reticular formation and locus ceruleus in the brainstem, respectively [11], [12]. These descending systems act on both presynaptic and postsynaptic sites to control the gain of neuronal excitability in nociceptive transmission [13], [14]. There is considerable evidence that the monoamines can act non-synaptically [15]. In the spinal and medullary dorsal horns, neurokinin 1 (NK1) receptor (SP receptor)-like immunoreactivities have been found principally in laminae I and III [16], from which the major pathways relaying noxious information to the thalamus, namely the spinothalamic tract and trigeminothalamic tract, originate [1]. These results suggest that NK1 receptor-containing neurons in the superficial laminae might receive nociceptive information conveyed by SP-containing primary afferent fibers and transmit it to the thalamus, thus fall into the projection neurons category.

The MDH is cytoarchitecturally and functionally similar to the spinal dorsal horn [1], but the circuity study in the MDH is very much limited compared with that in the spinal dorsal horn. Functional studies have revealed that GABA, Gly, 5-HT and NE are mainly involved in the antinociceptive effects [6], [17], [18], [19], however, there has been little evidence on the connections which they formed with (NK-1R-LI) projection neurons. Therefore, the present study was performed to elucidate the relationship by immunohistochemical dual stainings for GABA-, Gly-, 5-HT- or NE-containing terminals and NK1 receptor-like immunoreactive (LI) neurons in the MDH.

## Materials and Methods

Twenty adult male Wistar rats (weighing 250–300 g) were used in the present study. The Ethics Committee for Animal Experiments of the Fourth Military Medical University (Xi'an, P. R. China) approved all animal work (Permit number: 10001). According to the guidelines of the International Association for the Study of Pain (Zimmermann, 1983), all efforts were made to minimize the number of animals used and their suffering. During all surgical procedures, the rats were anesthetized by intraperitoneal injection of sodium pentobarbital (45 mg/kg) dissolved in 0.9% (w/v) saline.

### Immunofluorescence histochemistry

Ten rats were perfused transcardially with 150 ml of 0.01 M phosphate buffered-saline (PBS, pH 7.4) followed by 500 ml of 4% paraformaldehyde in 0.1 M phosphate buffer (PB, pH 7.4). The brainstems were removed and postfixed in the same fixative for 2 hours at 4°C. They were then cryoprotected in 30% sucrose in 0.1 M PB overnight at 4°C. The lower medulla oblongata was cut into frontal sections at 30 µm thickness with a freezing microtome (Kryostat 1720; Leitz, Mannheim, Germany). All sections were washed briefly with 0.01 M PBS and divided into six groups. The sections in the first dish were mounted onto gelatin-coated slides and stained lightly with 5% Neutral Red to observe the cytoarchitecture and to identify the boundaries of MDH laminae. Sections from the second to fifth dishes were incubated with one of the following antibody mixtures at room temperature overnight: (1) rabbit against GABA antiserum (1 µg/ml, A2052; Sigma, St. Louis, MO) and guinea-pig against NK1 receptor antiserum (1 µg/ml, AB5800; Chemicon, Temecula, CA); (2) rabbit against Gly antiserum (1 µg/ml, AB139; Chemicon) and guinea-pig against NK1 receptor antiserum (1 µg/ml; Chemicon); (3) rabbit against 5-HT antiserum (0.3 µg/ml, 20080; DiaSorin, Stillwater, MN) and guinea-pig against NK1 receptor antiserum (1 µg/ml; Chemicon); (4) rabbit against DBH antiserum (0.5 µg/ml, AB1585; Chemicon) and guinea-pig against NK1 receptor antiserum (1 µg/ml; Chemicon). The antibodies were diluted to their working concentrations in 0.01 M PBS containing 5% (v/v) normal goat serum (NGS), 0.3% (v/v) Triton X-100, 0.05% (w/v) NaN3 and 0.25% (w/v) carrageenan (PBS-NGS, pH 7.4). After rinsing three times in PBS, the slides were then incubated for 4 hours with biotinylated goat anti-rabbit IgG (1∶200 dilution; Vector, Burlingame, CA) in PBS-NGS. The slides were rinsed with PBS and then incubated with Alexa Fluor 488 conjugated goat anti-guinea pig IgG (1∶400 dilution; Molecular Probes, Eugene, Oregon), and Texas Red-labeled avidin D (1∶200 dilution; Vector) for 4 hours in PBS containing 0.3% Triton X-100. The slides were then rinsed in PBS and cover-slipped with a mixture of 5% (v/v) glycerin and 2.5% (w/v) triethylene diamine in 0.1 M PBS. The sections were observed with a confocal laser-scanning microscope (Fluoview 1000, Olympus, Tokyo, Japan), using laser beams of 543 and 488 nm with appropriate emission filters for Texas Red (590–610 nm) and Alexa 488 (510–525 nm), respectively. Digital images were captured using FLUOVIEW software (Olympus, Tokyo, Japan). The average diameters of NK1-immunopositive neurons were calculated by averaging the major diameter with the minor diameter; the major and minor diameters were the longest and shortest axes, respectively. A total of 96 neurons were measured in 10 animals to calculate the density of the different types of inputs contacting NK1 receptor-LI neurons per mm. The neurons measured were mostly located in lamina I. The density of inputs contacting NK1 receptor-LI neurons was measured for the entire soma for each cell and the proximal dendrites of the NK1 receptor-LI neurons. Proximal dendrites were defined as those within 30 µm of the soma. A mixture of normal rabbit and guinea pig sera was used to replace the first specific rabbit and guinea pig primary antibodies to incubate the sections from the sixth dish. The following staining procedures were same as those mentioned above. No immunopositive staining was found on these sections.

### Immuno-electron microscopy

Ten rats were anesthetized and perfused transcardially through the ascending aorta with 100 ml of 0.01 M PBS, followed by 500 ml of fixative containing 4% (w/v) paraformaldehyde, 0.05% (v/v) glutaraldehyde and 15% (v/v)-saturated picric acid in 0.1 M PB. The brains were removed and postfixed in the same fresh fixative for 4–6 hours at 4°C, and then put into 30% sucrose in 0.1 M PB (pH 7.4) overnight at 4°C. The lower medulla oblongata was cut into frontal sections at 50 µm thickness with a vibratome (Microslicer DTM-1000; Dosaka EM, Kyoto, Japan). All sections were washed briefly with 0.01 M PBS and divided into five groups.

Sections from the first to fourth dishes were collected in individual vials containing a mixture of 25% (w/v) sucrose and 10% (v/v) glycerol in 0.05 M PB (pH 7.4) for 30 minutes, respectively. In order to enhance the penetration of antibodies in the subsequent immunohistochemical staining procedures, the sections were freeze-thawed in liquid nitrogen. The sections were then washed three times in 0.05 M Tris-HCl buffered-saline (TBS; pH 7.4), incubated with 20% (v/v) normal goat serum in TBS for 30 minutes to block the non-specific immunoreactivity. Subsequently, sections first to fourth dishes were processed for immunohistochemical double-staining of GABA and NK1 receptor, Gly and NK1 receptor, 5-HT and NK1 receptor, or DBH and NK1 receptor according to the immunohistochemical double-staining procedures by using avidin-biotin-horseradish peroxidase complex (ABC) method for GABA, Gly, 5-HT and DBH and pre-embedding immuno-nanogold technique for NK1 receptor, respectively. Sections in each dish were incubated with one of the following mixtures of the antibodies respectively at room temperature overnight: (1) rabbit against GABA antiserum (1.5 µg/ml, A2052; Sigma, St. Louis, MO) and guinea-pig against NK1 receptor antiserum (1.5 µg/ml, AB5800; Chemicon, Temecula, CA); (2) rabbit against glycine antiserum (1.5 µg/ml, AB139; Chemicon) and guinea-pig against NK1 receptor antiserum (1.5 µg/ml; Chemicon); (3) rabbit against 5-HT antiserum (0.5 µg/ml, 20080; DiaSorin, Stillwater, MN) and guinea-pig against NK1 receptor antiserum (1.5 µg/ml; Chemicon); (4) rabbit against DBH antiserum (0.8 µg/ml, AB1585; Chemicon) and guinea-pig against NK1 receptor antiserum (1.5 µg/ml; Chemicon). The incubation medium was 0.05 M TBS containing 2% (v/v) normal goat serum (TBS-NGS, pH 7.4). After being washed with 0.05 M TBS (pH 7.4), the sections were incubated for 14–16 hours at room temperature with a mixture of goat antibody against guinea pig IgG conjugated to 1.4 nm gold particles (1∶100; Nanoprobes, Stony Brook, NY) and 10 µg/ml biotinylated donkey anti-rabbit IgG (Jackson Immuno Research, West Groove, PA) diluted with TBS-NGS (pH 7.4). Then all sections were washed with 0.01 M TBS (pH 7.4), post-fixed with 1% (w/v) glutaraldehyde in 0.1 M PB (pH 7.4) for 10 minutes and finally washed briefly with distilled water. Subsequently, silver enhancement was done in the dark with HQ Silver Kit (Nanoprobes). Then, the sections were incubated at room temperature with ABC Elite kit (1∶50; Vector, Burlingame, CA) in 0.05 M TBS (pH 7.4) for 3 hours. Furthermore, the sections were processed for peroxidase reaction. The sections were incubated at room temperature with 0.05 M Tris-HCl buffer (pH 7.6) containing 0.02% diaminobenzidine (DAB; Dojin, Kumamoto, Japan) and 0.0003% (v/v) H_2_O_2_ for 20–30 minutes, and then placed into 0.1 M PB (pH 7.4) containing 1% (w/v) OsO_4_ for 1 hour. Subsequently, the sections were counterstained with 1% (w/v) uranyl acetate in 70% ethanol for 1 hour. After dehydration, the sections were mounted on silicon-coated glass slides and flat embedded in epoxy resin (Durcupan; Fluka, Buchs, Switzerland). Once the resin had polymerized, the flat-embedded sections were examined under a dissection microscope. Four-to-five regions of the MDH that contained dense GABA-, Gly-, 5-HT- or DBH-LI terminals and NK1 receptor-LI neuronal cell bodies or dendritic processes were selected from each brain stem, and these small pieces of laminae I, II and III of the MDH were cut out with fresh razor blades. The samples of the selected tissue pieces were cut into 50–70-nm-thick ultrathin sections (silver sections) on an ultramicrotome (LKB, Bromma, Sweden). The ultrathin sections were mounted onto single-slot grids (6–8 sections per grid) coated with piloform membrane (Agar Scientific, Stansted, UK) and examined with an electron microscope (CM100, Philips, Eindhoven, the Netherlands). A total of 10–15 grids from each region were examined under the electron microscope and one or two grids were used for quantification. A total of 80 grids from different regions of the ten brain stems were for quantification. Electron micrographs were developed in the dark room and were not computer processed.

A mixture of normal rabbit and guinea pig sera was used to replace the first specific rabbit and guinea pig primary antibodies to incubate the sections from the fifth dish. The following staining procedures were same as those mentioned above. No immunopositive staining was found on the sections.

## Results

### Contacts revealed by immunofluroscent labeling

**Figure 1:**
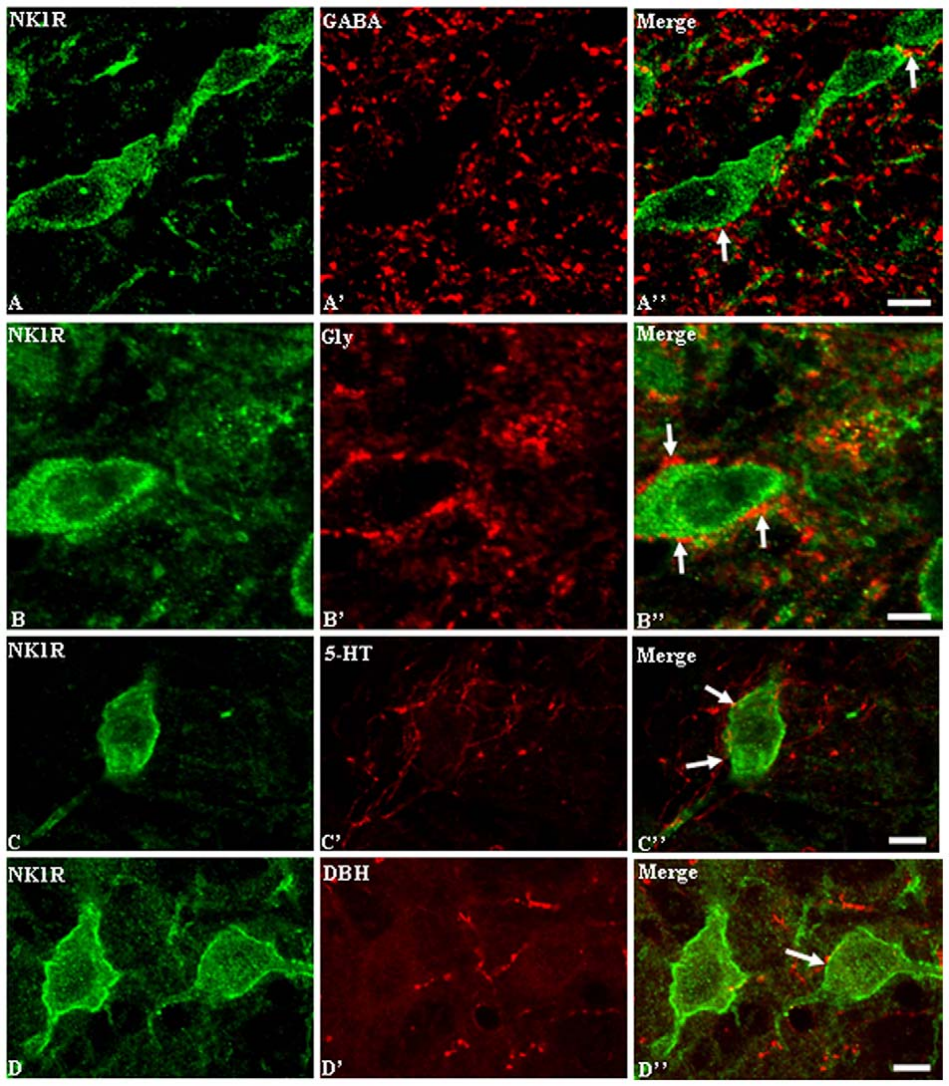
Immunofluorescent double labeling of γ-aminobutyric acid (GABA)-, glycine (Gly)-, serotonin (5-HT)- or dopamine-β-hydroxylase (DBH) with neurokinin 1 (NK1) receptor in the MDH of adult rats. NK1 receptor immunoreactivity is mainly distributed along the cellular and dendritic membrane of the MDH neurons (green), GABA, Gly, 5-HT or DBH - ir boutons (red) are in close apposition to NK1R-ir somas and dendrite (arrows). Scale bars, 10 µm ( A–D, A′–D′, A″–D″).

Many NK1 receptor-like immunoreactive (LI) neurons with well-developed dendritic processes were observed in the medullary dorsal horn (MDH). In the MDH, NK1 receptor-LI neurons were often oval, fusiform, or irregular in shape, and different in size. Most of the immunoreactive cells with soma areas >200 µm^2^. The NK1 receptor-immunoreactivity products were distributed mainly along the plasma membrane of somas and contiguous primary dendrites (Fig. 1 A, B, C, D). Most NK1 receptor-LI neurons were located in laminae I and III. The dendrites of NK1 receptor-LI neurons in lamina I ramify mainly within lamina I, some of them extended ventrally into laminae II and III. In lamina II, only a few NK1 receptor-LI neurons were sparsely encountered, whereas the dendrites of these NK1 receptor-LI neurons extended laterally into lamina I and/or ventrally into lamina III. Some large NK1 receptor-LI neurons were found in lamina III with their long dendritic processes extending dorsally into lamina II and some of them even occasionally extending into lamina I.

**Figure 2:**
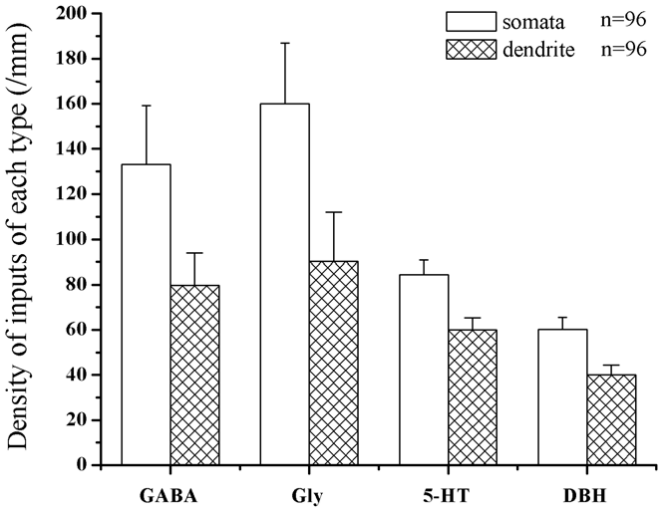
The density of the different types of inputs contacted with NK1 receptor-LI neurons ( /mm). Histogram showing input to NK1 receptor-LI somata and dendrites obtained with the confocal microscope.

GABA-, Gly-, 5-HT- and DBH-LI fibers and terminals were densely encountered in laminae I, II and III, especially in lamina II. A few GABA- and Gly-LI neuronal cell bodies were located principally in lamina II. Quite long 5-HT-LI and DBH-LI fibers covered with many bead-like varicosities were also observed in laminae I, II and III (Fig. 1 A′, B′, C′, D′). Some of the GABA-, Gly-, 5-HT- and DBH-LI boutons were closely apposed to the membrane of NK1 receptor-LI somata and dendrites (Fig. 1A″, B″, C″, D″). We observed 96 NK1 receptor-LI neurons contacting GABA-, Gly-, 5-HT- and DBH - LI terminals obtained with the confocal microscope in a stack of images at different (z) planes. The average number of the different types of terminals in contact with NK1 receptor-LI somata and dendrites per mm were calculated. The range of the number of contacts seen per cell was >200 µm^2^, and the length of proximal dendrites analysed was within 30 µm of the soma. Overall, the density of GABA-, Gly-, 5-HT- or DBH-LI terminals in contact with NK1 receptor-LI somata was higher than the density of contacts on dendrites (Fig. 2), and the densities of contacts from monoamine-containing axons were fewer than those from GABA or glycine axons (Fig. 2).

### Synaptic connections revealed with immuno-electron microscopy

**Figure 3:**
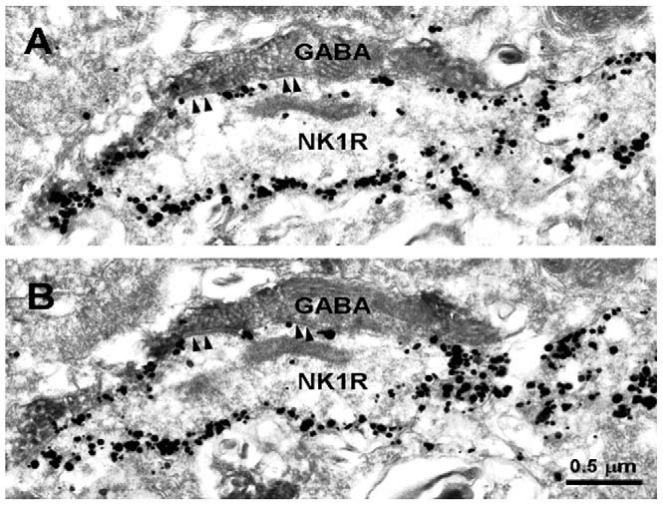
Electron micrographs of two adjacent sections, showing a GABA-immunoreactive axon terminal (GABA) visualized by peroxidase immunoreaction products that makes symmetric synapses with a NK1R-immunopositive dendrite (NK1R) revealed by silver grains in the MDH. Arrowheads indicate symmetric post-synaptic dense substance (PSD).

**Figure 4:**
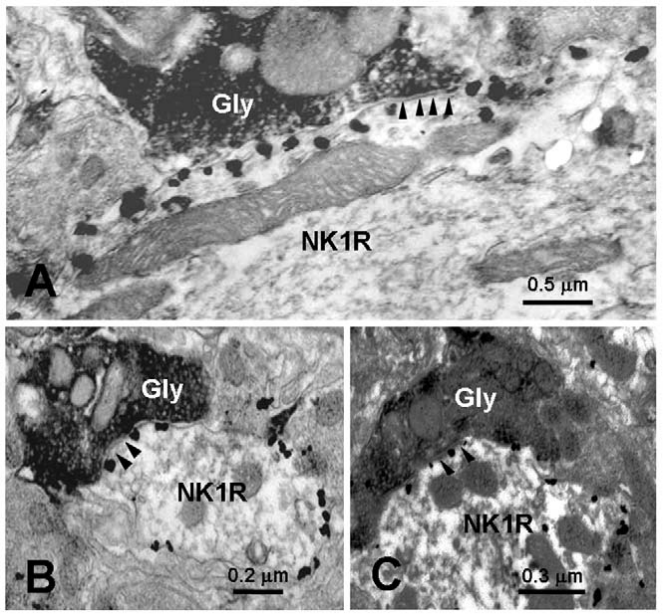
Electron micrographs showing the Gly-immunoreactive axon terminals (Gly) visualized by peroxidase immunoreaction products making symmetric synapses with NK1R-immunopositive cell body (NK1R; A) or dendrite (NK1R; B and C) revealed by silver grains in the MDH, respectively. Arrowheads indicate symmetric post-synaptic dense substance (PSD).

**Figure 5:**
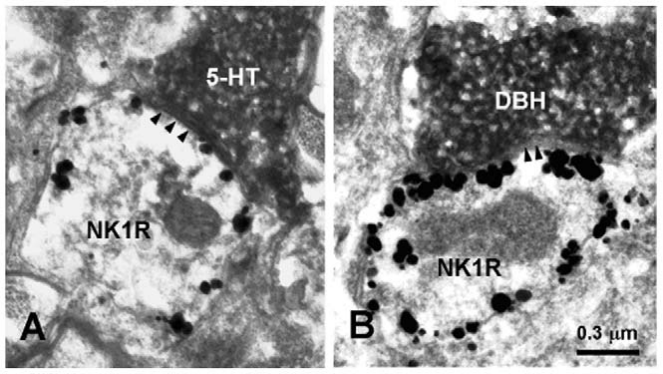
Electron micrographs showing a serotonin -immunoreactive axon terminal (5-HT; A) and a dopamine-β-hydroxylase-immunopositive axon terminal (DBH; B) visualized by peroxidase immunoreaction products making symmetric synapses with two NK1R-immunopositive dendrites (NK1R) revealed by silver grains in the MDH, respectively. Arrowheads indicate symmetric post-synaptic dense substance (PSD).

Electron microscopy revealed that typical nano-gold-labeled products, after enhancement with the HQ Silver Kit, were black round or oval particles with high electron densities and usually found underneath the plasma membrane of the cell bodies and large dendritic processes of neurons, which indicated the location of NK1 receptor (Figs. 3, 4, 5). GABA-, Gly-, 5-HT- and DBH-LI axonal terminals, usually filled with synaptic vesicles, were characterized by the presence of electron dense DAB reaction products adhering to the outer surface of cell organelles such as mitochondria, synaptic vesicles and the inner surface of the plasma membrane.

Axonal terminals exhibiting GABA- or Gly-like immunoreactivities were usually filled with pleomorphic synaptic vesicles in which the flattened synaptic vesicles were predominant and made symmetric synaptic contacts with dendritic processes and neuronal somatic profiles labeled with nano-gold particles which indicated the location of the NK1 receptor. There is no marked difference between the thickening of the pre- and post- synaptic membranes. In laminae I, II and III, GABA-LI terminals were found making synaptic connections with somatic profiles and dendritic processes (Fig. 3) of NK1 receptor-LI neurons. One hundred and twenty one synapses made by GABA-LI terminals were observed (Table 1), of which, 97% (117/121) were symmetric synapses and the rest 3% (4/121) were asymmetric ones. The majority of these synapse made by GABA-containing terminals and somatic profiles (21%, 25/121) or dendritic processes (79%, 96/121) of the NK1 receptor-LI neurons were found in lamina I, only a few of them were found in laminae II and III (Table 1).

**Table 1:** Synaptic types between GABA-, glycine-, 5-HT- and DBH-immunoreactive terminals and NK1R-immunopositive somatic profiles and dendritic processes in MDH.

	NK1R (+) somatic profiles		NK1R (+) dendritic processes		Total	
	Symmetric synapses	Asymmetric synapses	Symmetric synapses	Asymmetric synapses	Symmetric synapses	Asymmetric synapses
GABA (+)	24 (20%)	1 (1%)	93 (77%)	3 (2%)	117 (97%)	4 (3%)
Glycine (+)	11 (14%)	1 (1%)	67 (83%)	2 (2%)	78 (96%)	3 (4%)
5-HT (+)	10 (16%)	0 (0%)	50 (78%)	4 (6%)	60 (94%)	4 (6%)
DBH (+)	8 (21%)	1 (3%)	27 (71%)	2 (5%)	35 (92%)	3 (8%)

Gly-LI terminals were located throughout laminae I, II and III, but they were highest density in lamina II. Gly-LI axonal terminals were occasionally found to form synaptic connections with NK1 receptor-LI neuronal somatic profiles (Fig. 4a) and frequently with dendritic profiles (Fig. 4b). We also found 81 Gly-LI terminals in synaptic contacts with NK1 receptor-containing somatic profiles and dendritic processes (Table 1), of which, 96% (78/81) were symmetric synapses, and the rest 4% (3/81) were asymmetric ones. The majority of these synapse made by Gly-containing terminals and somatic profiles (15%, 12/81) or dendritic processes (85%, 69/81) of the NK1 receptor-LI neurons were found in lamina I, only a few of them were found in laminae II and III (Table 1).

**Figure 6:**
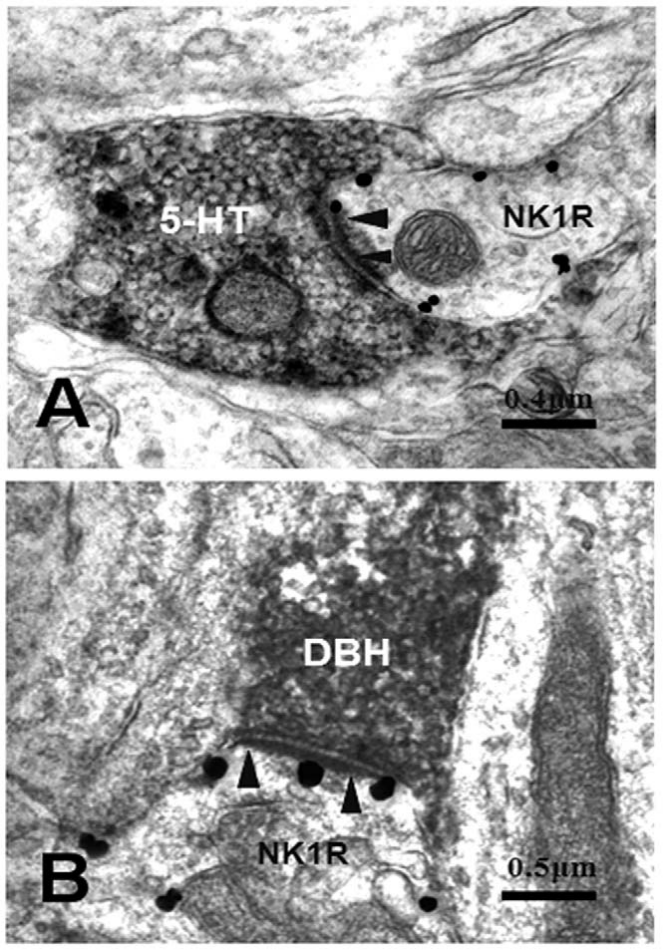
Electron micrographs showing a serotonin -immunoreactive axon terminal (5-HT; A) and a dopamine-β-hydroxylase-immunopositive axon terminal (DBH; B) visualized by peroxidase immunoreaction products making asymmetric synapses with two NK1R-immunopositive dendrites (NK1R) revealed by silver grains in the MDH, respectively. Arrowheads indicate synaptic active zones.

In the MDH, 5-HT- or DBH-LI were only found in axonal processes and their terminals. Axonal terminals exhibiting 5-HT- or DBH-like immunoreactivities were usually filled with pleomorphic synaptic vesicles in which the small spherical synaptic vesicles were predominant and made symmetric synaptic contacts with NK1 receptor-LI dendritic processes and somatic profiles. In laminae I, II and III, 5-HT-LI terminals were found to make synaptic connections with somatic profiles and dendritic processes (Fig. 5a) of NK1 receptor-LI neurons. Sixty four synapses made by 5-HT-LI terminals were observed (Table 1), of which, 94% (60/64) were symmetric synapses and the rest 6% (4/64) were asymmetric synapses. The thickening and density is much more pronounced in the post- than pre-synaptic membrane. Such synapses are designated asymmetric synapses (Fig. 6). The majority of these synapses made by 5-HT-containing terminals and NK1 receptor-LI neurons were found in lamina I, only a few of them were found in laminae II and III .

DBH-LI terminals were principally observed in lamina I and sparsely found in laminae II and III. DBH-LI axonal terminals were occasionally found to form synaptic connections with NK1 receptor-LI somatic profiles and frequently with dendritic profiles (Fig. 5b). We also found 38 DBH-LI terminals in synaptic contacts with NK1 receptor-containing somatic profiles and dendritic processes (Table 1), of which, 92% (35/38) were symmetric synapses (Fig. 4), and the rest 8% (3/38) were asymmetric synapses (Fig. 6).

## Discussion

### NK1 receptor-containing neurons in the MDH and NK1 receptor system involved in nociceptive transmission

After the antiserum against NK1 receptor was raised from rabbit [16], many studies have been carried out, especially on the distributions [16], fiber connections [6], [20], [21]–[24], and chemical natures [25] of the NK1 receptor-containing neurons. NK1 receptor is expressed predominantly in neurons of the central nervous system (CNS). In the rat MDH, neurons with intense NK1 receptor-immunoreactivities are located in laminae I and III [16]. The NK1 receptor is associated with projection neurons, it is also expressed by interneurons in the dorsal horn, and therefore it is not certain that the NK1 receptor-immunoreactive dendrites and cell bodies examined in this study belong to projection neurons. In fact, a recent study [26] has reported that in the rat lumbar spinal cord, cells in lamina I with a high level of NK1 receptor expression are probably all projection cells. Since the level of expression of the receptor is likely to be similar on cell bodies and dendrites of individual neurons, the dendrites that we observed, and which presumably had strong NK1 receptor expression, were likely to have originated from projection neurons. And NK1 receptor-immunoreactive cell bodies were likely to belong to projection neurons.

NK1 receptors are distributed throughout the cellular and dendritic membrane of neurons, at which SP containing primary afferent fibers make synaptic contacts [3], [4], [27]. NK1 receptors are activated by SP released following noxious stimuli and ablation of NK1 receptor-containing neurons with a substance P–saporin conjugate prevents the development of hyperalgesia [28], [29]. It has been suggested that NK1 receptor-LI neurons within superficial layers of the spinal dorsal horn play a critical role in transmiting and enhancing nociception [25]. Previous evidence has shown that most NK1 receptor-LI neurons show receptor internalization following noxious stimuli [30], which is important for intracellular signaling of nociception.

### Local inhibitory inputs to NK1 receptor-LI projection neurons in the MDH

MDH is involved principally in orofacial nociceptive transmission to higher brain structures, including thalamic regions [31]. The incoming information is processed by complex circuits involving inhibitory interneurons, and is transmitted to projection neurons for relaying to several brain areas [6]. Despite the importance of NK1 receptor-LI projection neurons in processing pain, the synaptic connections of the NK1 receptor-LI neurons in the MDH are still poorly understood. Studies have demonstrated that NK1 receptor-LI projection neurons in lamina I and lamina III in the spinal dorsal horn are densely innervated by peptidergic primary afferents, most of which contain SP [6]. In the present study, we found that NK1 receptor-LI neurons in the MDH received GABAergic and glycinergic inputs. These electron microscopic data have provided direct morphological evidence that GABA- and Gly-containing inhibitory neurons are involved in regulating the activities of NK1 receptor-LI neurons in processing orofacial nociceptive information.

GABA as one major classical inhibitory transmitter, has been observed in neurons, particularly within interneurons, fibers and terminals in the superficial part of the dorsal horn [6]. Local inhibitory interneurons in the MDH are significantly important in controlling the flow of nociceptive information and blocking inhibitory transmission in the spinal dorsal horn can lead to allodynia [32]. The present study has demonstrated that a large number of GABA- (97%) and Gly (96%)-LI boutons make symmetric synapses with NK1 receptor-LI somata or dendrites. These represent the morphological bases for inhibitory inputs to the NK1 receptor-LI projection neurons. It indicates that inhibitory local interneurons modulate orofacial nociceptive transmission. GABA can produce both presynaptic inhibition of primary afferents through axoaxonic synapses and postsynaptic inhibition of neurons mediated by axosomatic and axodendritic synapses [33]. In vivo study demonstrated that NK1 receptor-LI projection neurons had been under strong GABAergic control [34] and injection of the selective GABA_A_ receptor antagonist is sufficient to produce hyperalgesia [32], [35]. We speculate that NK1 receptor-LI projection neurons might be modulated by GABA-containing axons from interneurons via GABA_A_ receptor by postsynaptic inhibition [32], [35], [36]. In addition, GABA-containing axons which originate from RVM also project to the dorsal horn [37], [38].

Gly is another major inhibitory neurotransmitter in the CNS. It acts on the strychnine-sensitive glycine receptors (GlyR). GlyR are pentameric chloride channel composed of α and β subunits [39], [40]. Activation of GlyR generates inhibitory postsynaptic potentials (IPSPs). Administration of GlyR antagonists produces behavioural signs of allodynia [32], [35]. Some lamina I projection neurons that express gephyrin, a GlyR α1 and β subunit-associated protein, are either devoid of NK1 receptor or expressing NK1 receptors at a very low level [41]. These projection neurons are under powerful inhibitory control presumably from glycinergic local neurons. In this study, we found that NK1 receptor-LI neurons also received glycine input and previous study suggested a distinct expression of GlyRα3 in the superficial dorsal horn where nociceptive afferents terminate [39]. Thus, we speculate that the Gly which act on NK1 receptor-LI projection neurons is mediated at least partly via GlyR α3 subunits. Some GABAergic neurons in the superficial dorsal horn neurons are enriched with Gly-like immunoreactivity [6], and these are thought to use Gly as a co-transmitter. It is possible that GABA and Gly co-released at the same time from the same axonal terminal to act on NK1 receptor-LI projection neurons.

### Descending pain-modulating inputs to NK1 receptor-LI neurons in the MDH

In addition to local excitatory and inhibitory inputs, NK1 receptor-LI projection neurons also received descending serotonin (5-HT)- or norepinephrine (NE)-containing inputs from the brainstem. It has been indicated that orofacial nociceptive transmission is also modulated by these inputs. In the CNS, serotoninergic inputs come mainly from raphe nuclei, and norepinephrinergic inputs originate from locus ceruleus in the brainstem, respectively, but 5-HT- and NE-containing fibers and terminals are localized throughout the CNS [11], [12]. The descending control systems are involved in nociceptive and antinociceptive circuits [36]. Descending control systems alter the behavior of projection neurons in several ways: 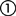 synapsing directly on the projection neurons; 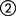 influencing the primary afferent inputs; 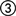 modulating interneurons' activities [36]. We have found that a large number of 5-HT-LI terminals (94%) and NE-LI terminals (92%) contact or synapse with NK1R-LI somata and dendrites. These findings are morphological evidence that the descending control systems exert their function via direct synapse on project neurons in the MDH.

5-HT-LI terminals mainly originate from the RVM, including NRM and its surrounding reticular formation [12]. Serotonergic descending pathways from brainstem form an endogenous analgesic system. Electrophysiological data demonstrated that 5-HT induced inhibition on SP-sensitive (putative NK-1 receptor expressing) dorsal horn neurons in vitro [42]. We have found that 5-HT-LI terminals contact with NK1 receptor-LI somata and dendrites in the superficial dorsal horn. 5-HT exerts its actions through binding with 5-HT_1_ to 5-HT_7_ receptors. Most of these receptors are G protein-coupled receptors. The 5-HT_3_ receptor subtype is a voltage-dependent cation channel complex [43]. The 5-HT_3_ receptor subtype involves in facilitating nociceptive pain via disinhibitory mechanisms. The 5-HT_5a_ receptor involves in stimulation-produced opioid analgesia via postsynaptic mechanism [36]. Moreover, 5-HT_1_ and/or 5-HT_7_ receptors which are expressed on the fibers are also involved in modulating nociceptive transmission [36]. More effort should be put in elucidating which 5-HT receptor subtye(s) is (are) involved in mediating serotonin function on to NK1 receptor LI projection neurons.

NE-containing neuronal cell bodies are encountered principally in the locus ceruleus underneath the fourth ventricle at the caudal pontine region [44]. Its receptors are divided into two main categories, alpha- and beta-adrenoceptors. Alpha-adrenoceptors are divided into subtypes 1A, 1B, 1D, 2A, 2B, and beta-adrenoceptors into subtypes1–3 [44]. Pharmacological studies indicated that administration of various synthetic adrenoceptor agonists produced different effects on pain responses [44]. Alpha-2A-adrenoceptor is located on primary afferent terminals and involves in the control of pain through presynaptic inhibition [36], [44]. Alpha-2-adrenoceptor is present on pain-relay neurons and involves in nociceptive information through postsynaptic inhibition. Alpha-1-adrenoceptor involves in activation of GABAergic and glycinergic inhibitory interneurons and alpha-2C-adrenoceptors in the dorsal horn may also contribute to pain regulation [44]. Our results have showed that the NE descending system directly synapsed on NK1 receptor-LI projection neurons. Further investigations are required to elucidate which subclass of receptors are involved in this procedure.

There have been studies in the spinal dorsal horn showing synapses from axons containing GABA [45], norepinephrine [46] or 5-HT [47] on projection neurons in lamina I, however, our results have demonstrated that neurons with NK1 receptor-immunoreactivity received not only local GABAergic and Glycinergic inhibitory inputs from interneurons but also descending 5-HT- or NE-containing inputs from brainstem in laminae I, II and III of the MDH. The density of GABA-, Gly-, 5-HT- or DBH-LI terminals in contact with NK1 receptor-LI somata was different from the density of contacts on dendrites, and this may reflect functional differences. It therefore provides direct anatomical evidence and quantitative study within the MDH. Previous studies have demonstrated that GABAergic and Glycinergic neurons in the rat MDH could be activated directly by SP-fibers [48]. SP containing primary afferent fibers also make synaptic contacts with neurons with NK1 receptor-immunoreactivity. Thus, NK1 receptor-LI neurons might be an important, but not the exclusive player in orofacial nociceptive and antinociceptive circuits. However, there is relatively little information about NK1 receptor-LI neurons that express other receptors and ion channels. Further investigations should focus on identifying their neurochemical features.
